# Rasch analysis of the self efficacy (SE-12) questionnaire measuring clinical communication skills

**DOI:** 10.1016/j.pecinn.2024.100296

**Published:** 2024-05-28

**Authors:** Kaj Sparle Christensen, Jette Ammentorp

**Affiliations:** a Research Unit for General Practice, Aarhus, Denmark; b Department of Public Health, Aarhus University, Denmark; c Centre for Research in Patient Communication, Odense University Hospital of Southern, Denmark, Odense; d Department of Clinical Research, University of Southern Denmark, Odense, Denmark

**Keywords:** Self efficacy, SE-12, Rasch analysis

## Abstract

**Objective:**

The aim of this study was to examine the construct validity and reliability of the Self Efficacy (SE-12) questionnaire using Rasch analysis.

**Methods:**

The SE-12 was administered to Danish health care professionals prior to their participation in a communication skills training program. Analysis of fit to the Rasch model, ordering of response categories, dimensionality-testing, test for differential item functioning, test for local dependency, and calculation of reliability were used to evaluate the psychometric characteristics of the SE-12.

**Results:**

In this study, 1057 respondents were analyzed. Overall, the SE-12 demonstrated an acceptable fit to the Rasch model. Response categories were appropriately ordered for all twelve items. However, items 6 (structure) and item 8 (empathy) demonstrated differential item functioning, with men being more likely to affirm the first and women the last item. Local dependency was observed between five item groups but adjusting for these improved fit indices significantly. The SE-12 exhibited high reliability with PSI ranging from 0.92 to 0.94. A transformation table converting ordinal scores to interval scores is provided.

**Conclusion:**

The SE-12 demonstrates good construct validity and excellent reliability. Minor issues regarding local dependency and differential functioning require attention.

Innovation: A 5-item version could be explored without compromising validity and reliability.

## Highlights

• The SE-12 demonstrates robust construct validity and high reliability.
• However, it is crucial to address gender-based differences and item dependency.
• There is potential for a shorter and more concise version of the SE-12.
• Researchers are encouraged to use the provided transformation table.

## 1 Introduction

Effective patient-centered communication is crucial for ensuring high-quality healthcare delivery. To equip healthcare professionals with the necessary communication skills, it is essential to assess the effectiveness of communication training programs [[1], [2], [3]]. However, measuring the effectiveness of such programs can be challenging, and there is a growing demand for validated measures that accurately reflect the skills learned [4]. This importance is highlighted by research demonstrating the ongoing challenge of transferring communication skills to clinical practice [[5], [6], [7]], but also by evidence indicating that evidence-based methods for training and measuring effects can address these challenges [8].

One widely used measure to assess the impact of communication skills training is self-efficacy [[9], [10], [11], [12], [13], [14], [15], [16], [17], [18]]. Self-efficacy, a key element of social cognitive theory [19], refers to an individual's confidence in their ability to successfully perform a specific behavior or task [20]. Research has shown that self-efficacy plays a predictive and mediating role in motivation, learning, and performance [21].

Self-efficacy scales have been developed to assess an individual's level of self-efficacy in specific domains, and they typically consist of a set of items or statements that individuals rate on a scale from strongly disagree to strongly agree. For example, a typical item on an academic self-efficacy scale might be, “I am confident that I can successfully complete challenging academic tasks.”

The self-efficacy scale SE-12 [22] was developed to measure the impact of communication skills training. It is based on the well-established construct of self-efficacy and was developed through pilot studies at Department of Pediatrics and at Medical School [9,23]. Since then, the SE-12 has been employed to evaluate to measure the outcomes of mandatory communication skills training for healthcare professionals at a Danish hospital [17]. Furthermore, it has been in high demand, attracting numerous personal requests to the authors seeking permission to use it in various international research and quality development projects.

Although the SE-12 questionnaire has been widely used in healthcare, limited research has been conducted on its psychometric properties, particularly regarding its dimensionality and item functioning [22].

Rasch analysis is a modern psychometric technique, that can be used to investigate the properties of self-efficacy measures, such as the SE-12. It addresses the inherent limitations of using ordinal scale sum scores, which, while indicative of change, fail to accurately quantify the magnitude of such changes due to variable item difficulties and the non-linear nature of response categories. By transforming ordinal data into interval-level measurements, Rasch analysis provides a detailed assessment of a measure's validity, dimensionality, and reliability. This method provides a more accurate and meaningful analysis, facilitating the identification of nuanced changes in self-efficacy [[24], [25], [26], [27]].

Therefore, the aim of this study was to examine the construct validity and reliability of the SE-12 questionnaire using Rasch analysis.

## 2 Methods

### 2.1 Sample

The evaluation of the SE-12 is based on data from a study of a communication skills training program for health care professionals in Denmark [17,28]. In order to ensure a diverse range of SE-12 scores, we used data prior to course participation (Q1).

### 2.2 Statistical approach

#### 2.2.1 Rasch measurement model analysis

Following the guidelines for reporting Rasch Measurement (RM) Theory studies in rehabilitation research (RULER) [29,30], our analyses were performed using Rasch Unidimensional Measurement Model (RUMM2030) [31] and Stata [32].

#### 2.2.2 Development and description of the SE-12

The development of the SE-12 was originally developed through focus group discussions with communication experts and participants in a communication skills course for clinicians. The scale comprises twelve items, each evaluated on a 10-point scale, assessing respondents' confidence in various communication skills [22].

#### 2.2.3 Model assessment requirements

Respondents are automatically placed into groups called class intervals of approximately equivalent sizes across the sample. An initial likelihood ratio test was performed to determine the most appropriate mathematical derivation of the Rasch model for the data set. If a significant result (*p* < 0.05) is obtained, it will support the use of the unrestricted partial credit model rather than the simpler rating scale model.

#### 2.2.4 Internal consistency and item fit analysis

Initially, an estimation of the internal consistency of the scale was used using the person separation index (PSI). The PSI is analogous to Cronbach's alpha. A number of tests were applied to the SE-12 to examine how well it conformed to the Rasch model. Test of individual item fit to the Rasch model reflect the differences between the observed responses and that expected by the model. These tests are presented for each item as a fit residual and as a chi-square probability statistic. A residual is a summation of individual item (or person) deviations from model expectations, which are then standardized to form a z-score. Residual scores between ±2.5 are considered to generally indicate adequate fit to the model [33]. The chi-square statistic tests if the difference between the observed and expected values across the class interval for each item are statistically significant or not. A non-significant chi-square statistic >0.05, or Bonferroni adjusted value to account for multiple testing, indicates good fit to the model [33].

#### 2.2.5 Threshold ordering and dimensionality

The twelve SE-12 items each has a polytomous response format ranging from 1 to 10. It would be expected that as self-efficacy rises, it is more likely to score a 1, then a 2, then a 3, and so on, until 10 on any particular item. In Rasch analysis terms, this would be indicated by an ordered set of response thresholds for each item [33]. Disordered thresholds can occur when respondents have difficulty consistently discriminating between response options. In such options it is common practice to collapse disordered response categories with the aim of improving the alignment with the Rasch model an enhancing overall fit.

To classify a construct being measured as unidimensional and justify a total summated score, there must be one prominent factor underlying it. Two subsets of items are identified by testing the factor loadings on the first principal component of the residuals. The highest positive set of correlated items are tested against the highest negative set of correlated items using *t*-tests to determine if they significantly differ from each other [34]. If the person severity estimate is found to significantly differ in >5% of respondents, this would indicate that the two subsets are so different that they measure different, but possibly related constructs. A confidence interval is then applied and its lower bound should overlap 5% for a non-significant test [35].

#### 2.2.6 Differential item functioning and local dependence

A possible source of misfit in the data is differential item functioning (DIF). This is a type of bias such as when different respondents in the sample respond in a different manner to an item despite equally levels of self-efficacy. DIF was tested using analysis of variance (ANOVA) and a statistically significant probability *p* < 0.05 (or the Bonferroni adjusted level) indicates a potential DIF problem. Local dependency (LD) was tested to determine whether the response to any item had a direct impact to the response to any other item. A cut point of 0.20 above average residual correlation was considered significant [36].

#### 2.2.7 Scale targeting

Targeting of the questionnaire items to the population facilitates the identification of potential gaps in item difficulty, informing further scale refinement.

## 3 Results

### 3.1 Sample characterization

Sample demographics are displayed in Table 1.

**Table 1:** Sample demographics (*n* = 1057).

Demographic		N (%)
Gender	Male	115 (10.88)
	Female	942 (89.12)
Age, years	<30	128 (13.62)
	30–39	255 (27.13)
	40–49	268 (28.51)
	50–59	232 (24.68)
	≥60	57 (6.06)
Profession	Physician	143 (13.53)
	Nurse	703 (66.51)
	Other⁎	221 (19.96)
Specialty	Surgery	316 (29.90)
	Medicine	666 (63.01)
	Therapy	75 (7.10)

⁎ ) Physio therapist, Occupational therapist, and Midwife.

### 3.2 Fit to the Rasch model

In total, 6 persons had extreme scores (all responding 0 to all items) and were excluded from further analysis as no information was provided on rank ordering of persons and items. The unrestricted partial credit model was used.

The initial analysis revealed a nonsignificant item-trait interaction statistic (*χ*2(108) = 132.95, *p* = 0.05), which indicated fit to the Rasch model (Table 2, analysis 1). The summary fit residual for items (2.12) and fit residuals for persons (1.63) were both within acceptable limits (±2.5). However, among the 1057 individual studied, 14.9% showed fit residuals above +/− 2.5.

**Table 2:** Model fit statistics for SE-12 scale items.

Action	Analysis	Model fit (overall)	Item fit residual, mean (SD)	Person fit residual, mean (SD)	PSI	Unidimensionality, significant t-tests (CI95%)	Local dependency (residual correlations >0.20 above average)
Original sample (*n* = 1051)	1	*χ*2(108) =132.95, *p* = 0.052	0.23 (2.12)	−0.56 (1.63)	0.94	12.11 (10.86–13.50)	Items 1&2&6, 3&5, 4&7&8, 9&10, 11&12
Adjusted sample (*n* = 500)	2	*χ*2(108) =63.25, *p* = 0.999	0.23 (2.12)	−0.56 (1.63)	0.94	12.11 (10.86–13.50)	Items 1&2&6, 3&5, 4&7&8, 9&10, 11&12
Subtest analysis, collapse of items 1&2&6, 3&5, 4&7&8, 9&10, 11&12	3	*χ*2(45) =55.81, *p* = 0.130	0.14 (2.62)	−0.51 (1.21)	0.92	7.54 (6.22–8.86)	–

*χ*2: chi-square; p: probability; SD: standard deviation; PSI: person separation index (with extremes).

The *χ*2 value is likely to have been affected by the large sample size (*n* > 500), making even minor deviations from the Rasch model statistically significant [37,38]. When the overall *χ*2 was adjusted based on a sample of 500 respondents (available in RUMM2030), no overall misfit was detected, *p* = 0.98 (Table 2, analysis 2).

The initial analysis of fit statistics for individual items is presented in Table 3. It appears, that Item 9 (fit residual −3.171) and item 10 (fit residual −2.606) exhibit slight over discrimination, while item 4 (fit residual 3.477) shows slight underdiscrimination.

**Table 3:** Individual item fit statistics for SE-12 items (n = 1051).⁎

Item	Loocation	Fit residual	*χ*^2^	*P*robability*
	
1 How certain are you that you are able to successfully identify the issues the patient wishes to address during the conversation?	0.254	1.345	13.078	0.160
2 How certain are you that you are able to successfully make an agenda/plan for the conversation with the patient?	0.491	3.477	10.564	0.307
3 How certain are you that you are able to successfully urge the patient to expand on his or her problems/worries?	--0.123	0.219	8.699	0.465
4 How certain are you that you are able to successfully listen attentively without interrupting or changing of focus?	−0.338	1.671	7.944	0.539
5 How certain are you that you are able to successfully encourage the patient to express thoughts and feelings?	−0.109	−0.633	6.277	0.712
6 How certain are you that you are able to successfully structure the conversation with the patient?	0.453	2.173	15.912	0.069
7 How certain are you that you are able to successfully demonstrate appropriate non-verbal behavior (eye contact, facial expression, placement, posture, and voicing)?	0.007	2.320	18.223	0.033
8 How certain are you that you are able to successfully show empathy (acknowledge the patient's views and feelings)?	−0.401	0.938	7.697	0.565
9 How certain are you that you are able to successfully clarify what the patient knows in order to communicate the right amount of information?	0.059	−3.171	16.779	0.052
10 How certain are you that you are able to successfully check patient's understanding of the information given?	−0.145	−2.606	9.743	0.372
11 How certain are you that you are able to successfully make a plan based on shared decisions between you and the patient?	−0.036	−0.898	11.696	0.231
12 How certain are you that you are able to successfully close the conversation by assuring, that the patient's questions have been answered?	−0.062	−2.083	6.337	0.706

⁎ Bonferroni-adjusted at 1% level.

### 3.3 Adequacy of response categories

Inspection of the category probability curves demonstrated ordered thresholds for all 12 items (data not shown).

### 3.4 Local dependency

Initial analyses indicated local dependency among specific items (1&2&6, 3&5, 4&7&8, 9&10, 11&12) in the dataset. To address this, we combined the dependent items into five testlets (super-items) in the analysis (Table 2, analysis 3), which improved the overall fit to the Rasch model. A table demonstrating the integration of SE-12 items into super-items is displayed in the supplementary.

### 3.5 Dimensionality testing

Testing for dimensionality was performed by comparing the positive and negative factor-loading items of the first component of the principal component analysis of residuals. The analyses revealed significant *t*-tests outside the critical value of 5%, indicating that the total SE-12 severity score measure is not a single unidimensional construct (Table 2 and 4).

However, by combining the dependent items into five separate testlets, a clear improvement was observed (Table 2, analysis 3).

To further address the dimensionality issue, we performed a confirmatory factor analysis (CFA) of a five-factor model, incorporating the five testlets. The CFA demonstrated that the five-factor model (Χ^2^ = 189.84, RMSEA (90% CI) = 0.056 (0.048–0.064), CFI = 0.982, TLI = 0.973) provided a better fit compared to the simpler one-factor model (Χ ^2^ = 7.12.68, RMSEA (90% CI) = 0.107 (0.100–0.115), CFI = 0.920, TLI = 0.902).

### 3.6 Differential item functioning

**Fig. 1:**
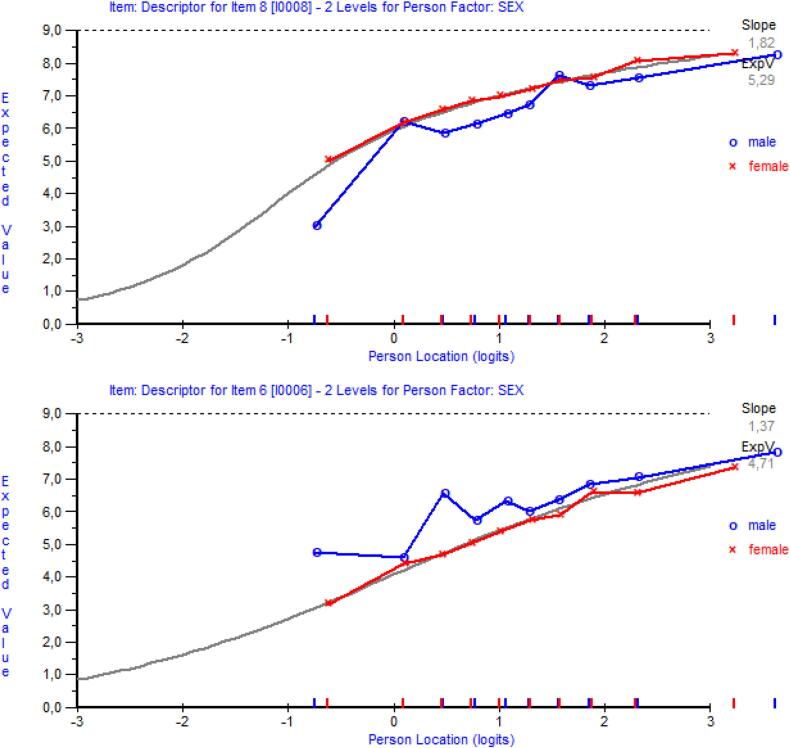
Item 6 and item 8 displaying Differential Item Functioning regarding sex.

Item 6, “able to successfully structure” and item 8, “able to successfully show empathy” demonstrated uniform DIF with male respondents being more likely to affirm the first item and less likely to affirm the latter item than female respondents (Fig. 1).

### 3.7 Targeting of the scale

**Fig. 2:**
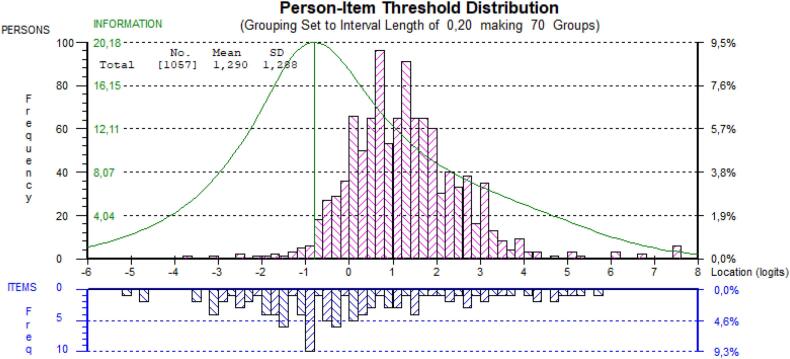
Person-item threshold distribution displaying the relative logit distribution of the SE-12 item thresholds and the population sample.

The person-item distribution map (Fig. 2) showed that respondents had a mean location of 1.29 logits with a standard deviation of 1.29, indicating suboptimal targeting. However, the items were well distributed along the difficulty continuum. The easiest item to endorse was item 8, “able to successfully show empathy”, while the hardest item to endorse was item 2, “successfully make an agenda”. A Wright map is provided in the supplementary.

### 3.8 Reliability

The person separation reliability (PSI) was high (0.94) and sufficient for distinguishing between individual persons. However, when we combined dependent items into five testlets, there was a slight decline in PSI to 0.92.

### 3.9 Transformation table

A transformation table, included in the supplementary materials, allows for converting ordinal summary scores to interval scores for both genders. The table takes into account the issues related to local dependency and differential functioning of items, ensuring a more accurate interpretation of the results.

## 4 Discussion and conclusion

### 4.1 Discussion

The SE-12 demonstrated an acceptable fit to the Rasch model expectations in this sample; however, measurement problems were identified in terms of local dependency and differential item functioning of single items. These local dependencies clearly violated the unidimensionality assumptions of the instrument.

By grouping the dependent items into five testlets; items 1&2&6 (issues, agenda, structure), items 3&5 (worries, thoughts and feelings), items 4&7&8 (focus, behavior, empathy), items 9&10 (information, understanding), items 11&12 (plan, closure) clearly improved the model fit without compromising reliability. This reconfiguration points towards the possibility of condensing the SE-12 into a more focused, 5-item version, emphasizing key communication skills for clinicians: 1) identifying problems and structuring the conversation, 2) exploring concerns, 3) maintaining focus and empathy, 4) achieving understanding, and 5) planning and closure. This streamlined version requires field testing to affirm its validity and reliability.

Furthermore, the differential functioning issues for items on “structure” (item 6) and “empathy” (item 9) were addressed by implementing gender-specific summary scores. This approach aligns with literature on gender differences, suggesting that such differences may have evolved due to distinct societal roles [39]. This finding stresses the need for gender-specific contexts in the evaluation of communication skills, to properly account for historical and evolutionary influences on behavior.

These modifications have strengthened the SE-12's measurement properties, which is crucial for its effective application in research and practice. In our initial study of the SE-12, we employed a classical test theoretical approach, which indicated that the instrument was likely unidimensional [22]. However, the recent findings challenge this assumption, revealing that the SE-12 comprises five distinct factors or clusters of highly related items. Consequently, we urge future research to address this problem, possibly by rephrasing the interdependent items.

Findings from this study apply to the actual sample of respondents only. Generalizing the findings from this Rasch analysis to different populations or settings require caution and, ideally, additional validation studies.

### 4.2 Innovation

There is a possibility that a 5-item version of the SE-12 could be developed without loss of reliability and validity. This simpler version focuses on the core of effective clinical communication, possibly making it easier to evaluate and enhance how clinicians communicate with their patients. A proposal for how to formulate the SE-5 can be found in the table of SE-12 domains in the supplementary materials.

### 4.3 Conclusion

The SE-12 demonstrates good construct validity and excellent reliability. Minor issues regarding local dependency and differential item functioning require attention. A 5-item version could be explored without compromising validity and reliability. Meanwhile, we recommend researchers to utilize the provided transformation table converting ordinal scores to interval scores until further improvements are made. The transformation table effectively addresses concerns related to dependency and differential functioning of items, enhancing the precision of interpreting SE-12 summary scores. By addressing these issues, we can further strengthen the overall utility and effectiveness of the SE-12 as a valuable assessment tool for communication skills in healthcare professionals.

## Ethics approval

This study was a secondary analysis. Human subjects were not involved, and identifiable information was not included.

## Funding

This research did not receive any specific grant from funding agencies in the public, commercial, or not-for-profit sectors.

## CRediT authorship contribution statement

**Kaj Sparle Christensen:** Writing – review & editing, Writing – original draft, Methodology, Formal analysis, Conceptualization. **Jette Ammentorp:** Writing – review & editing, Writing – original draft, Resources, Investigation.

## Declaration of competing interest

The authors declare that they have no known competing financial interests or personal relationships that could have appeared to influence the work reported in this paper.

## Appendix A Supplementary data

Supplementary material. Image 1
