# Supplementary material for: Rasch analysis of the self efficacy (SE-12) questionnaire measuring clinical communication skills
Source: PEC Innov. 2024 May 28;4:100296. doi: 10.1016/j.pecinn.2024.100296 (PMC11169456; doi:10.1016/j.pecinn.2024.100296)
Supplement: Supplementary file 1 — Supplementary material. [file mmc1.docx]

**Table of SE-12 domains identified.**

| Testlets (super-items) | SE-12 items | Content | Proposed SE-5 items |
| --- | --- | --- | --- |
| 1&2&6 | 1. How certain are you that you are able to successfully identify the issues the patient wishes to address during the conversation?  2. How certain are you that you are able to successfully make an agenda/plan for the conversation with the patient?  6. How certain are you that you are able to successfully structure the conversation with the patient? | Being able to identify issues, make an agenda, and to structure the conversation. | 1. How confident are you in your ability to successfully identify issues, create an agenda, and structure the conversation with the patient? |
| 3&5 | 3. How certain are you that you are able to successfully urge the patient to expand on his or her problems/worries?  5. How certain are you that you are able to successfully encourage the patient to express thoughts and feelings? | Being able to expand on problems or worries, to encourage the patient to express thoughts and feelings. | 2. How confident are you in your ability to successfully expand on problems or worries, and to encourage the patient to express thoughts and feelings |
| 4&7&8 | 4. How certain are you that you are able to successfully listen attentively without interrupting or changing of focus?  7. How certain are you that you are able to successfully demonstrate appropriate non-verbal behavior (eye contact, facial expression, placement, posture, and voicing)?  8. How certain are you that you are able to successfully show empathy (acknowledge the patient’s views and feelings)? | Being able to listen attentively, show appropriate nonverbal behavior and empathy | 3. How confident are you in your ability to listen attentively, display suitable nonverbal behavior, and demonstrate empathy? |
| 9&10 | 9. How certain are you that you are able to successfully clarify what the patient knows in order to communicate the right amount of information?  10. How certain are you that you are able to successfully check patient’s understanding of the information given? | Being able to clarify what the patient knows and understands. | 4. How confident are you in your ability to clarify what the patient knows and understands? |
| 11&12 | 11. How certain are you that you are able to successfully make a plan based on shared decisions between you and the patient?  12. How certain are you that you are able to successfully close the conversation by assuring, that the patient’s questions have been answered? | Being able to make a plan based on shared decisions and close the conversation by assuring that the patient’s questions have been answered. | 5. How confident are you in your ability to collaboratively create a plan and conclude the conversation, ensuring all the patient's questions have been addressed? |

**Wright map. Compares individual abilities against the difficulties of test items on a common scale expressed in logits.**

**
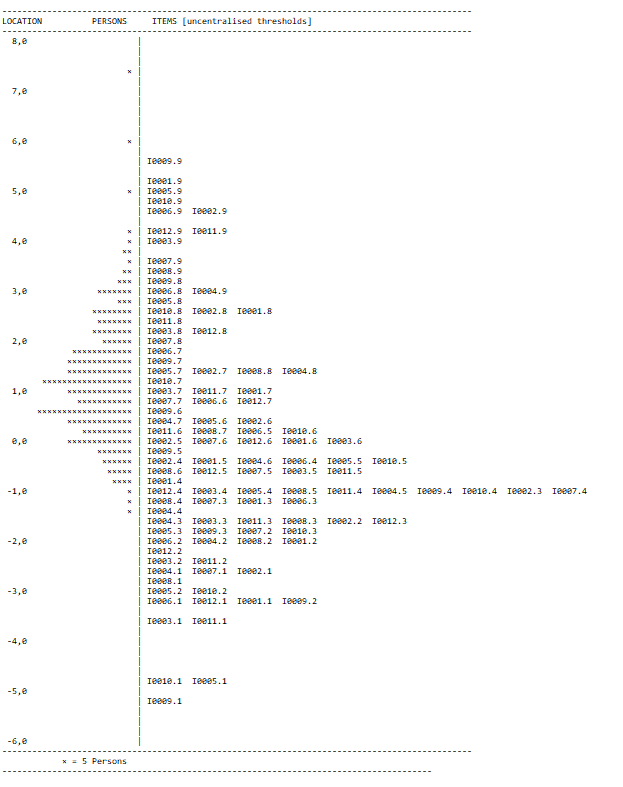
**

**Transformation table for SE-12. Conversion of ordinal summary scores to interval scores for both genders.**

|  | Female | | Male | |
| --- | --- | --- | --- | --- |
| Ordinal score | Location in logits | Interval score | Location in logits | Interval score |
| 12 | -5,53 | 12,00 | -4,10 | 12,00 |
| 13 | -4,72 | 19,49 | -3,44 | 19,08 |
| 14 | -4,17 | 24,56 | -3,02 | 23,58 |
| 15 | -3,80 | 27,97 | -2,75 | 26,45 |
| 16 | -3,52 | 30,57 | -2,56 | 28,55 |
| 17 | -3,29 | 32,67 | -2,40 | 30,23 |
| 18 | -3,10 | 34,45 | -2,27 | 31,63 |
| 19 | -2,93 | 35,99 | -2,16 | 32,83 |
| 20 | -2,79 | 37,35 | -2,06 | 33,88 |
| 21 | -2,66 | 38,57 | -1,98 | 34,82 |
| 22 | -2,54 | 39,68 | -1,90 | 35,67 |
| 23 | -2,43 | 40,70 | -1,83 | 36,43 |
| 24 | -2,32 | 41,63 | -1,76 | 37,14 |
| 25 | -2,23 | 42,50 | -1,70 | 37,78 |
| 26 | -2,14 | 43,32 | -1,65 | 38,38 |
| 27 | -2,06 | 44,07 | -1,59 | 38,93 |
| 28 | -1,98 | 44,79 | -1,55 | 39,44 |
| 29 | -1,91 | 45,46 | -1,50 | 39,93 |
| 30 | -1,84 | 46,10 | -1,46 | 40,38 |
| 31 | -1,78 | 46,71 | -1,42 | 40,81 |
| 32 | -1,71 | 47,30 | -1,38 | 41,22 |
| 33 | -1,65 | 47,86 | -1,35 | 41,61 |
| 34 | -1,59 | 48,41 | -1,31 | 41,98 |
| 35 | -1,54 | 48,93 | -1,28 | 42,34 |
| 36 | -1,48 | 49,43 | -1,25 | 42,68 |
| 37 | -1,43 | 49,92 | -1,21 | 43,02 |
| 38 | -1,38 | 50,41 | -1,18 | 43,34 |
| 39 | -1,33 | 50,87 | -1,16 | 43,65 |
| 40 | -1,28 | 51,32 | -1,13 | 43,95 |
| 41 | -1,23 | 51,77 | -1,10 | 44,25 |
| 42 | -1,18 | 52,20 | -1,07 | 44,55 |
| 43 | -1,14 | 52,63 | -1,05 | 44,83 |
| 44 | -1,09 | 53,04 | -1,02 | 45,10 |
| 45 | -1,05 | 53,46 | -1,00 | 45,37 |
| 46 | -1,00 | 53,86 | -0,97 | 45,64 |
| 47 | -0,96 | 54,26 | -0,95 | 45,90 |
| 48 | -0,92 | 54,65 | -0,92 | 46,16 |
| 49 | -0,88 | 55,04 | -0,90 | 46,41 |
| 50 | -0,83 | 55,43 | -0,88 | 46,67 |
| 51 | -0,79 | 55,81 | -0,85 | 46,91 |
| 52 | -0,75 | 56,19 | -0,83 | 47,16 |
| 53 | -0,71 | 56,57 | -0,81 | 47,41 |
| 54 | -0,67 | 56,94 | -0,78 | 47,66 |
| 55 | -0,63 | 57,31 | -0,76 | 47,91 |
| 56 | -0,59 | 57,68 | -0,74 | 48,16 |
| 57 | -0,55 | 58,05 | -0,71 | 48,42 |
| 58 | -0,51 | 58,42 | -0,69 | 48,68 |
| 59 | -0,47 | 58,78 | -0,66 | 48,94 |
| 60 | -0,43 | 59,15 | -0,64 | 49,21 |
| 61 | -0,39 | 59,52 | -0,61 | 49,49 |
| 62 | -0,35 | 59,89 | -0,59 | 49,77 |
| 63 | -0,31 | 60,26 | -0,56 | 50,06 |
| 64 | -0,27 | 60,63 | -0,53 | 50,35 |
| 65 | -0,23 | 61,00 | -0,50 | 50,66 |
| 66 | -0,19 | 61,37 | -0,47 | 50,98 |
| 67 | -0,15 | 61,75 | -0,44 | 51,30 |
| 68 | -0,11 | 62,13 | -0,41 | 51,65 |
| 69 | -0,07 | 62,52 | -0,38 | 52,00 |
| 70 | -0,03 | 62,91 | -0,35 | 52,37 |
| 71 | 0,02 | 63,29 | -0,31 | 52,76 |
| 72 | 0,06 | 63,69 | -0,27 | 53,16 |
| 73 | 0,10 | 64,10 | -0,23 | 53,57 |
| 74 | 0,15 | 64,51 | -0,19 | 54,01 |
| 75 | 0,19 | 64,92 | -0,15 | 54,46 |
| 76 | 0,24 | 65,35 | -0,11 | 54,93 |
| 77 | 0,29 | 65,77 | -0,06 | 55,42 |
| 78 | 0,33 | 66,22 | -0,02 | 55,92 |
| 79 | 0,38 | 66,67 | 0,03 | 56,45 |
| 80 | 0,43 | 67,13 | 0,09 | 57,00 |
| 81 | 0,48 | 67,61 | 0,14 | 57,57 |
| 82 | 0,54 | 68,10 | 0,19 | 58,15 |
| 83 | 0,59 | 68,60 | 0,25 | 58,76 |
| 84 | 0,65 | 69,11 | 0,31 | 59,40 |
| 85 | 0,70 | 69,64 | 0,37 | 60,06 |
| 86 | 0,76 | 70,19 | 0,43 | 60,74 |
| 87 | 0,82 | 70,75 | 0,50 | 61,45 |
| 88 | 0,89 | 71,32 | 0,57 | 62,18 |
| 89 | 0,95 | 71,93 | 0,64 | 62,95 |
| 90 | 1,02 | 72,54 | 0,71 | 63,73 |
| 91 | 1,09 | 73,18 | 0,79 | 64,55 |
| 92 | 1,16 | 73,83 | 0,87 | 65,39 |
| 93 | 1,23 | 74,52 | 0,95 | 66,26 |
| 94 | 1,31 | 75,22 | 1,03 | 67,17 |
| 95 | 1,38 | 75,94 | 1,12 | 68,09 |
| 96 | 1,47 | 76,69 | 1,21 | 69,06 |
| 97 | 1,55 | 77,47 | 1,30 | 70,05 |
| 98 | 1,64 | 78,27 | 1,39 | 71,07 |
| 99 | 1,73 | 79,10 | 1,49 | 72,13 |
| 100 | 1,82 | 79,96 | 1,59 | 73,22 |
| 101 | 1,91 | 80,85 | 1,70 | 74,35 |
| 102 | 2,01 | 81,76 | 1,80 | 75,50 |
| 103 | 2,12 | 82,71 | 1,92 | 76,69 |
| 104 | 2,22 | 83,70 | 2,03 | 77,92 |
| 105 | 2,34 | 84,74 | 2,15 | 79,19 |
| 106 | 2,45 | 85,81 | 2,27 | 80,50 |
| 107 | 2,57 | 86,94 | 2,40 | 81,87 |
| 108 | 2,70 | 88,13 | 2,53 | 83,28 |
| 109 | 2,84 | 89,38 | 2,66 | 84,74 |
| 110 | 2,98 | 90,71 | 2,81 | 86,28 |
| 111 | 3,13 | 92,12 | 2,96 | 87,92 |
| 112 | 3,30 | 93,64 | 3,12 | 89,64 |
| 113 | 3,48 | 95,29 | 3,29 | 91,50 |
| 114 | 3,67 | 97,08 | 3,48 | 93,55 |
| 115 | 3,89 | 99,09 | 3,69 | 95,82 |
| 116 | 4,13 | 101,38 | 3,94 | 98,43 |
| 117 | 4,43 | 104,12 | 4,23 | 101,56 |
| 118 | 4,81 | 107,61 | 4,60 | 105,60 |
| 119 | 5,35 | 112,67 | 5,15 | 111,47 |
| 120 | 6,15 | 120,00 | 5,94 | 120,00 |

Instruction: To use a transformation table, find your total score in the table, then look for the matching interval score next to it. This interval score is what you use for detailed analysis.
